# Predicting antibiotic susceptibility in urinary tract infection with artificial intelligence—model performance in a multi-centre cohort

**DOI:** 10.1093/jacamr/dlae121

**Published:** 2024-08-07

**Authors:** Alfred Lok Hang Lee, Curtis Chun Kit To, Ronald Cheong Kin Chan, Janus Siu Him Wong, Grace Chung Yan Lui, Ingrid Yu Ying Cheung, Viola Chi Ying Chow, Christopher Koon Chi Lai, Margaret Ip, Raymond Wai Man Lai

**Affiliations:** Department of Microbiology, Prince of Wales Hospital, Shatin, Hong Kong SAR, China; Department of Anatomical and Cellular Pathology, Faculty of Medicine, Chinese University of Hong Kong, Shatin, Hong Kong SAR, China; Department of Anatomical and Cellular Pathology, Faculty of Medicine, Chinese University of Hong Kong, Shatin, Hong Kong SAR, China; Department of Orthopaedics and Traumatology, School of Clinical Medicine, LKS Faculty of Medicine, University of Hong Kong, Pokfulam, Hong Kong SAR, China; Department of Medicine and Therapeutics, Prince of Wales Hospital, Shatin, Hong Kong SAR, China; Department of Microbiology, Prince of Wales Hospital, Shatin, Hong Kong SAR, China; Department of Microbiology, Prince of Wales Hospital, Shatin, Hong Kong SAR, China; Department of Microbiology, Faculty of Medicine, Chinese University of Hong Kong, Shatin, Hong Kong SAR, China; Department of Microbiology, Faculty of Medicine, Chinese University of Hong Kong, Shatin, Hong Kong SAR, China; Chief Infection Control Officer Office, Hospital Authority, Kowloon, Hong Kong SAR, China

## Abstract

**Objective:**

To develop an artificial intelligence model to predict an antimicrobial susceptibility pattern in patients with urinary tract infection (UTI).

**Materials and methods:**

26 087 adult patients with culture-proven UTI during 2015–2020 from a university teaching hospital and three community hospitals in Hong Kong were included. Cases with asymptomatic bacteriuria (absence of diagnosis code of UTI, or absence of leucocytes in urine microscopy) were excluded. Patients from 2015 to 2019 were included in the training set, while patients from the year 2020 were included as the test set.

Three first-line antibiotics were chosen for prediction of susceptibility in the bacterial isolates causing UTI: namely nitrofurantoin, ciprofloxacin and amoxicillin-clavulanate. Baseline epidemiological factors, previous antimicrobial consumption, medical history and previous culture results were included as features. Logistic regression and random forest were applied to the dataset. Models were evaluated by F1-score and area under the curve-receiver operating characteristic (AUC-ROC).

**Results:**

Random forest was the best algorithm in predicting susceptibility of the three antibiotics (nitrofurantoin, amoxicillin-clavulanate and ciprofloxacin). The AUC-ROC values were 0.941, 0.939 and 0.937, respectively. The F1 scores were 0.938, 0.928 and 0.906 respectively.

**Conclusions:**

Random forest model may aid judicious empirical antibiotics use in UTI. Given the reasonable performance and accuracy, these accurate models may aid clinicians in choosing between different first-line antibiotics for UTI.

## Introduction

Antimicrobial resistance (AMR) is an emerging threat that jeopardizes global health and development. Driven by inappropriate antimicrobial prescription, AMR threatens therapeutic success with both existing and new antibiotics.

Urinary tract infection (UTI) is the commonest cause of bacterial infection in both community and hospital settings worldwide.^1^ As the major driver of antibiotics prescription, UTI is a key target for antimicrobial stewardship. These efforts include laboratory stewardship, educational seminar, clinical decision support tool, development of clinical guidelines, clinical audit or feedback session.^2–6^ These are often, however, resource heavy and unsustainable. The ‘one-size-fit-all’ approach of antibiotics guidelines cannot address individual patient factors either. Clinicians often face a dilemma between inadequate antimicrobial coverage and promoting drug resistance when guidelines do not cover all clinical scenarios.

Machine learning (ML) is a field of artificial intelligence that focuses on algorithms that improve with data input. The application of these ML algorithms in antimicrobial stewardship is gaining traction due to the availability of large healthcare datasets.^7–9^ Many ML algorithms have been proposed to streamline empirical antimicrobial use in UTI by predicting the most likely resistance patterns in urine culture.^10–12^ When integrated into clinical practice, these ML algorithms can advise clinicians on the narrowest spectrum effective antibiotic based on the patient’s health data.

## Objective

In this study, we aim to develop a ML algorithm for culture-proven UTI to predict susceptibility of bacterial isolates to three first-line antibiotics, namely nitrofurantoin, amoxicillin-clavulanate and ciprofloxacin.

## Materials and methods

### Subjects

This was an epidemiological study of a territory-wide database of the public hospital network comprising the New Territories East Cluster of Hong Kong SAR, China. This hospital network consisted of one university teaching hospital, two district general hospitals and affiliated clinics. The public healthcare system was responsible for 90% of healthcare in this locality, and this hospital cluster served a population of 2 million in the region.

Adult patients who attended these hospitals with positive urine cultures within the period between 1 January 2015 and 31 December 2020 were considered for the study. For patients with multiple episodes of UTI in the study period, only the first episode of UTI for each patient in the study period was included. This was to allow a more unbiased representation of the patient population.

### Inclusion criteria

Patients with a positive urine culture (culture colony count ≥10^4^ cfu/mL) and the following criteria were considered to have culture-proven UTI:

- presence of white blood cell (WBC) in urine microscopy, and
- s confirmed diagnosis of UTI in Clinical Management System (ICD-9 codes: 590, 595 and 599.0)

### Exclusion criteria

- age < 18 years old
- discordance of result between concomitant blood culture and urine culture within 3 days
- three or more bacterial morphotypes in urine culture, i.e. potential contamination
- culture positive for yeasts, viridans *Streptococcus* species, coagulase-negative *Staphylococcus* species except *Staphylococcus saprophyticus* and *Corynebacterium* species.

It is well-recognized that the diagnosis of UTI in elderly can be challenging owing to the increasingly atypical presentation with age and the potential lack of a comprehensive history. Bearing in mind the possibility of asymptomatic bacteriuria especially among catheterized patients, the study therefore only included patients receiving a confirmed diagnosis of UTI from the clinical team, instead of all patients with positive urine cultures. As the elderly form the bulk of medical admissions in the locality, the inclusion of catheter urine ensures that many elderly patients with acute pyelonephritis, who are often too frail to save mid-stream urine on their own, were included in the study.

**Figure 1.**
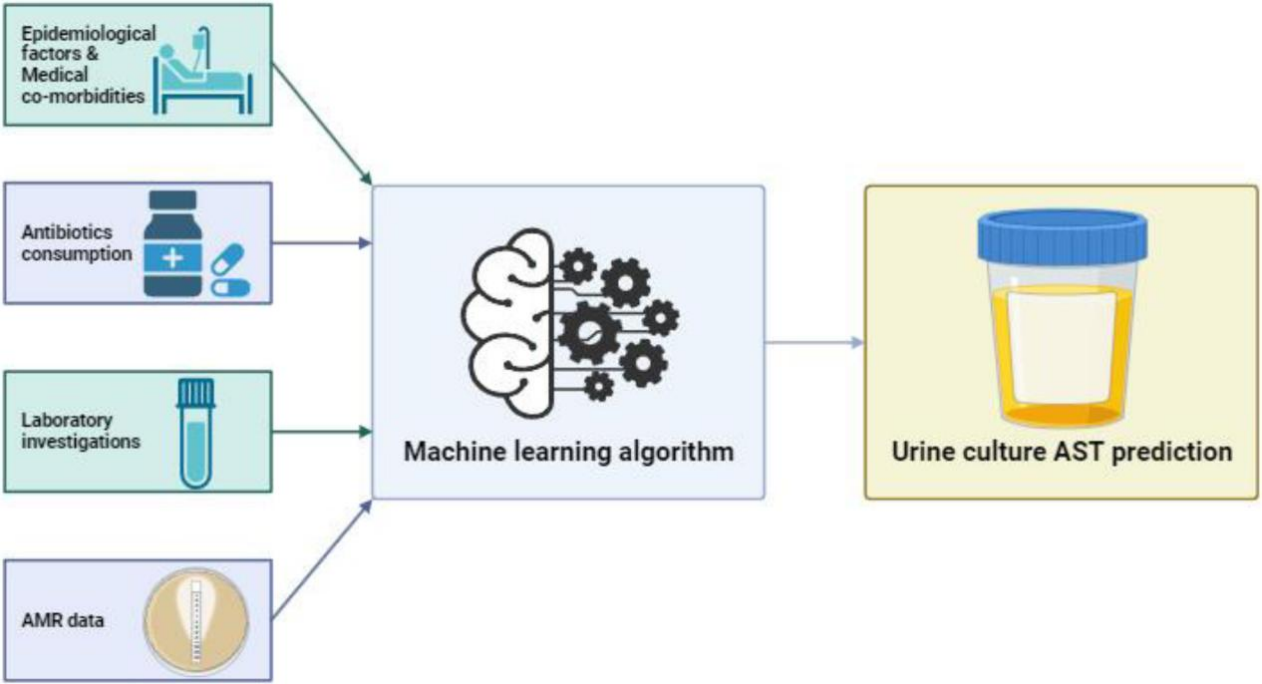
Study design.

Figure 1 illustrates the design of this study. Background healthcare data for 5 years before the episode of UTI (epidemiological factors, medical comorbidities, antibiotics consumption, laboratory investigations and AMR data) available on the study subjects before the episode of UTI were collected. For patients with multiple episodes of UTI during the study period, background data for 5 years before the first episode of UTI was included. Medical comorbidities were compiled as individual features. These were compiled as features (explanatory variables) and analysed with ML algorithms, for the prediction of antimicrobial susceptibility (AST) results in urine culture (labels or outcome variables).

### Data collection

Laboratory data were retrieved from Laboratory Information System of Microbiology laboratories in the hospital cluster. They included urine culture results and corresponding blood culture results of the proposed study period. For each specimen, laboratory data collected included collection date, cultured organism identity, antibiotic susceptibility pattern of each isolate, bacterial colony count and microscopy findings. UTI with concomitant bloodstream infection is not uncommon. For cases with bacteraemic UTI, combined sensitivity results based on both urine and blood culture were used for data analysis. The more resistant antibiogram was used in place of the more susceptible one.

Clinical data of the corresponding patients were retrieved through a territory-wide healthcare database, the Clinical Data Analysis and Reporting System. Medical records of these patients were screened for potential features, including age, gender, admission source, residence at old age home, previous in-patient stays, active diagnosis, antibiotic administration, culture results and procedures. Diagnoses were encoded according to International Classification of Diseases ninth revision Clinical Modification (ICD-9-CM). Medications other than antibiotics were grouped by the British National Formulary index. Antibiotics were grouped according to their class, with cephalosporins classified by generations. Blood investigation results were entered at both their raw numerical values and whether they were above or below the respective laboratory reference ranges.

For subjects with multiple urine culture specimens within the same episode of illness in the study period, the first urine specimen for that subject was included for prediction. Other subsequent urine culture data in the study period were excluded.

### Data analysis and machine learning

The dataset comprising of 6 years of urine culture result was split into a training set and a test set. The training set consisted of the data from 1 January 2015 to 31 December 2019, while the test set consisted of data from 1 January 2020 to 31 December 2020. R statistical language version 3.6.2 (R foundation, Vienna, Austria) was used for data analysis and ML.

Missing data were imputed by multiple imputation by chained equations method through the R package ‘mice’. For all classification methods except logistic regression, all features were normalized by minmax normalization to 0–1 range to ensure model convergence and optimization.

Exploratory data analysis and summary statistics were used to summarize the dataset. Variables with high correlation with others were omitted from the dataset. Each member of pairs of variables with correlation coefficient of 0.5 or above were removed with the choice of removal decided by subject domain knowledge.

In a preliminary analysis of model performance for different ML algorithms (XGBoost, decision tree, random forest, neural network, support vector machine) in the pilot study, random forest was found to be the best performing model. Hence optimization and further analysis of ML algorithm were focused on random forest. Logistic regression was included in this study as a comparator. Please refer to Table S1 (available as Supplementary data at *JAC-AMR* Online) for the full list of features used in the production of these models.

#### Logistic regression

Univariable logistic regression was performed for all explanatory variables. Model selection was done by a combination of expert domain knowledge input from the specialist in Clinical Microbiology and Infection, and bidirectional stepwise regression. The Hosmer–Lemeshow test was used to determine the goodness of fit. The logistic regression model was used to look for risk factors predicting antibiotics resistance in UTI. *P* values <0.05 were considered statistically significant.

#### Random forest

Decision tree models use a range of probabilistic rules arranged in the form of a flowchart to classify each observation into a variety of categories. The random forest model, as an ensemble method, uses many decision trees to optimize its predictive power. This was accessed through the R package ‘randomForest’. Intuitive explanation of random forest was performed by Shapley Additive Explanations (SHAP) values. This was accessed via the R package ‘treeshap’ with a unified representation of the random forest models. SHAP value of a feature represents the contribution of it to the predictive model.

#### Model evaluation

Model evaluation was done in the separate test set to ensure objectivity. Each prediction model was evaluated on the test set by sensitivity, specificity, positive predictive value (PPV), negative predictive value (NPV), macro-averaged F1 score and area under the curve-receiver operating characteristic (AUC-ROC).

### Ethical considerations

The declaration of Helsinki was followed in this study. Ethics approval was obtained for this study through the ethics committee (CREC reference number: 2022.628).

## Results

### Baseline epidemiological characteristics

A total of 72 276 positive urine culture samples were found in the period 2015–2020. After removing duplicate samples and non-UTI cases, a total of 26 087 patients were included in this study. Among these cases, 20 237 cases were from the period 2015–2019, and 5850 cases were from the year 2020.

In the data analysis, 105 features were included. Table 1 shows the baseline epidemiological characteristics of the cohort: 8888 (34.1%) of the cohort were male, and the mean age was 67.9 years. Most patients were given beta-lactam beta-lactamase-inhibitor combinations (mean defined daily dose (DDD) 40.71) or quinolones (mean DDD 10.22) in the past 5 years. For detailed description of background characteristics, please refer to Table S1.

**Table 1.** Baseline characteristics of the study cohort (*n* = 26 087)

Variable	Count (%)/mean (SD)
**(a) Antibiotic susceptibility of causative agent in the cohort**	
Nitrofurantoin	20 794 (79.7%)
Amoxicillin-clavulanate	18 658 (71.5%)
Ciprofloxacin	17 410 (66.8%)
**(b) Basic epidemiological factors**	
Male gender	8888 (34.1%)
Use of immunosuppressive within the past 5 years	2283 (8.75%)
Age in years	67.9 (21.0)
Patient source	
Emergency department	3277 (12.6%)
Hospital in-patient	14 303 (54.8%)
Clinic	8507 (32.6%)

Variable	Median (IQR)
**(c) Antibiotics consumption of each patient in the past 5 years (defined daily dose)**	
Beta-lactam beta-lactamase inhibitor combination	24 (48)
Carbapenems	0 (0)
Second-generation cephalosporins	0 (0)
Third-generation cephalosporins	0 (0)
Penicillins	0 (7)
Fluoroquinolones	0 (9)
Nitrofurantoin	0 (5)

Variable	Count (%)/mean (SD)
**(d) Blood test results in the previous 5 years before the index episode of UTI**	
Haemoglobin mean value (g/dL)	11.74 (1.86)
WBC mean value (×10^9^/L)	9.32 (3.89)
Platelet count mean value (×10^9^/L)	237.93 (85.55)
Creatinine mean value (μmol/L)	106.45 (97.14)
Bilirubin mean value (μmol/L)	12.08 (12.48)
Alanine transaminase mean value (IU/L)	27.94 (40.94)
Alkaline phosphatase mean value (IU/L)	96.81 (68.09)

Variable	Median (IQR)
**(e) Number of disease episodes in electronic health record in the previous 5 years**	
Diabetes mellitus (DM)	0 (1)
Gastrointestinal tract disorder	0 (2)
Genitourinary tract disorder	0 (1)
Number of episodes of infection	0 (1)
Retention of urine	0 (1)
Stroke	0 (2)
**(f) Percentage of patients with a urinary tract pathogen isolated in the past 5 years**	
Enterobacterales order	84.56
*Enterococcus* spp.	10.20
*Pseudomonas aeruginosa*	5.04
*Candida* spp.	3.40
Methicillin-susceptible *S. aureus* (MSSA)	1.78
MRSA	1.53
*Acinetobacter* spp.	1.03
*Streptococcus* spp.	0.96
Other organisms	2.06
**(g) Mean percentage of urinary tract bacterial pathogen susceptible to antibiotics in the past 5 years** ^a^	
Nitrofurantoin	76.54
Amoxicillin-clavulanate	68.55
Meropenem	91.03
Ciprofloxacin/Levofloxacin	62.82
Amikacin	99.53
Cefuroxime	65.99%
Cefotaxime/ceftriaxone	70.31%

^a^Further explanation for Table 1g: To capture the entire antimicrobial resistance pattern of each patient, we chose antibiotic susceptibility percentage of each patient as the summary statistic. For patients with only one isolate of urinary tract pathogen, this statistic would be based on that single isolate only. For example, if the patient only had 1 MSSA urinary tract isolate in the past 5 years, his/her amoxicillin-clavulanate percentage susceptibility would be 1/1 = 100%. Alternatively, if the patient had one isolate of MRSA only, then the amoxicillin-clavulanate percentage susceptibility would be 0/1 = 0%. For patients with multiple urinary tract pathogens isolated in past 5 years, this statistic would be calculated as an average proportion of organisms susceptible to the particular antibiotic. For example, if the patient had two isolates of MSSA and three isolates of MRSA in the past 5 years, the amoxicillin-clavulanate percentage susceptibility would be 2/5 = 40%. For patients without any urinary tract pathogen isolated in the past, the susceptibility percentage was considered 100%.

The distribution of causative agents in the current cohort are shown in Table 2.

**Table 2.** Distribution of causative agents of UTI in the study

Organism	In-patient	Community (via A&E)	Community (via clinic)
*E. coli*	8448	2347	5528
*Klebsiella* spp.	1661	262	964
*Enterococcus* spp.	1291	150	586
*P. mirabilis*	830	158	489
*P. aeruginosa*	505	52	100
*Citrobacter* spp.	301	85	247
*Enterobacter* spp.	281	60	131
*S. aureus*	420	50	148
*Morganella morganii*	196	38	118
*Serratia* spp.	73	10	34
Others	297	65	162

The commonest pathogen in the study was *Escherichia coli*, accounting for over half (62.6%) of cases. *Klebsiella* spp. (11.1%) and *Proteus mirabilis* (5.7%) were also commonly found. The percentage of extended-spectrum beta-lactamase (ESBL) resistance in Enterobacterales was 18.8% (3880/20 687).

### Logistic regression

Table 3 shows the performance of logistic regression for the three tested antibiotics.

**Table 3.** Performance of logistic regression in predicting antibiotic susceptibility

	Nitrofurantoin	Amoxicillin-clavulanate	Ciprofloxacin
Accuracy	0.909	0.895	0.874
Sensitivity	0.730	0.781	0.858
Specificity	0.952	0.938	0.884
F1 score	0.944	0.928	0.900
AUC-ROC	0.917	0.922	0.912
PPV	0.937	0.918	0.917
NPV	0.783	0.828	0.806

The AUC-ROC of logistic regression values for susceptibility prediction of nitrofurantoin, amoxicillin-clavulanate and ciprofloxacin were 0.917, 0.922 and 0.912, respectively. The F1 score of logistic regression for susceptibility prediction of nitrofurantoin, amoxicillin-clavulanate and ciprofloxacin were 0.944, 0.928 and 0.900, respectively.

**Figure 2.**
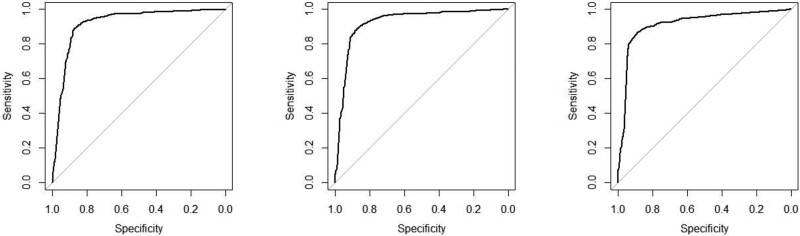
ROC curves of logistic regression models.

Figure 2 shows the ROC of logistic regression models. For precision-recall curves, please refer to Table S2.

Table 4 shows the performance and key predictive variables using logistic regression.

**Table 4.** Logistic regression model specifications

Logistic regression model with nitrofurantoin susceptibility as outcome variable		
Explanatory variable	Odds ratio (95% CI)	*P* values
Male gender	1.11 (0.98, 1.26)	0.09
Previous nitrofurantoin susceptibility in UTI	377.91 (320.23, 445.99)	<0.001
Previous amoxicillin-clavulanate susceptibility in UTI	3.91 (3.37, 4.53)	<0.001
Previous ceftriaxone susceptibility in UTI	0.22 (0.19, 0.26)	<0.001

Logistic regression model with amoxicillin-clavulanate susceptibility as outcome variable		
Explanatory variable	Odds ratio (95% CI)	*P* values
Previous ESBL-producing Enterobacterales in urine	0.24 (0.19, 0.3)	<0.001
Previous amoxicillin-clavulanate susceptibility in UTI	516.02 (437.08, 609.23)	<0.001
Previous ceftriaxone susceptibility in UTI	0.23 (0.18, 0.28)	<0.001

Logistic regression model with ciprofloxacin susceptibility as outcome variable		
Explanatory variable	Odds ratio (95% CI)	*P* values
Pulmonary disease	0.83 (0.77, 0.9)	<0.001
Previous MRSA	0.79 (0.73, 0.86)	<0.001
Previous Gram-positive organism in UTI	1.76 (1.45, 2.13)	<0.001
Previous nitrofurantoin susceptibility in UTI	0.46 (0.4, 0.53)	<0.001
Previous ciprofloxacin/Levofloxacin susceptibility in UTI	308.43 (267.21, 356)	<0.001
Previous Amikacin susceptibility in UTI	2.20 (1.01, 4.8)	0.0451
Previous ceftriaxone susceptibility in UTI	0.66 (0.57, 0.76)	<0.001

For prediction of nitrofurantoin susceptibility with logistic regression, statistically significant predictors were prior susceptibility to nitrofurantoin (OR 377.91, *P* < 0.001), amoxicillin-clavulanate (OR 3.91, *P* < 0.001) and ceftriaxone (OR 0.22, *P* < 0.001) in previous UTI pathogens.

For prediction of amoxicillin-clavulanate susceptibility with logistic regression, statistically significant predictors included previous ESBL-producing Enterobacterales in urine (OR 0.24, *P* < 0.001), previous susceptibility to amoxicillin-clavulanate (OR 516.02, *P* < 0.001) and ceftriaxone (OR 0.23, *P* < 0.001) susceptibility in UTI.

For prediction of amoxicillin-clavulanate susceptibility with logistic regression, statistically significant predictors included pulmonary disease (OR 0.83, *P* < 0.001), previous methicillin-resistant *Staphylococcus aureus* (MRSA) (OR 0.79, *P* < 0.001), previous Gram-positive organism in UTI (OR 1.76, *P* < 0.001) and previous susceptibility to nitrofurantoin (OR 0.46, *P* < 0.001), ciprofloxacin/levofloxacin (OR 308.43, *P* < 0.001), amikacin (OR 2.20, *P* = 0.045) and ceftriaxone (OR 0.66, *P* < 0.001) in UTI.

### Random forest

Table 5 shows the model performance for random forest. The AUC-ROC of random forest for susceptibility prediction of nitrofurantoin, amoxicillin-clavulanate and ciprofloxacin were 0.941, 0.939 and 0.937, respectively. The F1 score of logistic regression for susceptibility prediction of nitrofurantoin, amoxicillin-clavulanate and ciprofloxacin were 0.938, 0.928 and 0.906, respectively.

**Table 5.** Performance of random forest in predicting antibiotic susceptibility

	Nitrofurantoin	Amoxicillin-clavulanate	Ciprofloxacin
Accuracy	0.902	0.894	0.882
Sensitivity	0.840	0.765	0.860
Specificity	0.916	0.943	0.894
F1	0.938	0.928	0.906
AUC-ROC	0.941	0.939	0.937
PPV	0.960	0.913	0.919
NPV	0.705	0.837	0.820

**Figure 3.**
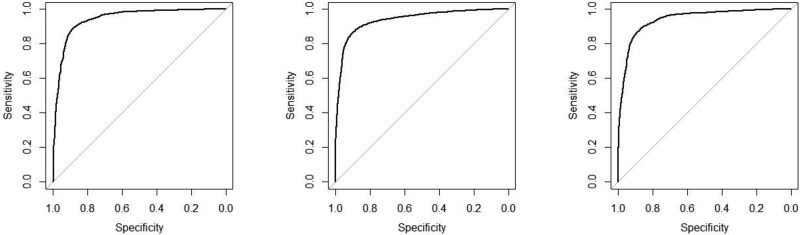
ROC curves of random forest models.

Figure 3 shows the ROC curves of random forest models.

**Figure 4.**
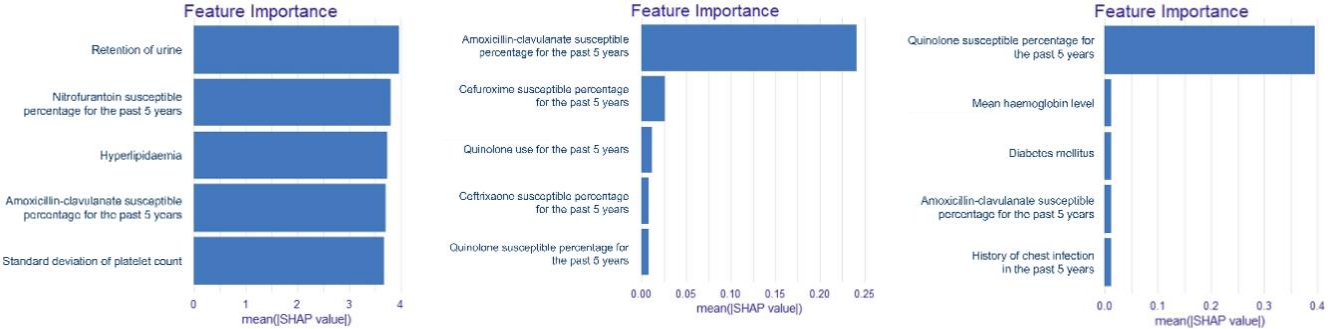
Features with the highest predictive power (SHAP value) in random forest models.

Five features with the highest predictive power, i.e. the highest SHAP values for each model (nitrofurantoin, amoxicillin-clavulanate, ciprofloxacin), are displayed in Figure 4.

The most important features (features with the highest SHAP values) in the nitrofurantoin model were urinary retention, previous nitrofurantoin susceptibility in UTI, hyperlipidaemia, previous amoxicillin-clavulanate susceptibility in UTI and the standard deviation of platelet count.

The most important features in the amoxicillin-clavulanate model was previous amoxicillin-clavulanate susceptibility in UTI. The single most important feature in the ciprofloxacin model was previous susceptibility to ciprofloxacin/levofloxacin in UTI.

## Discussion

The study evaluated the performance of various ML methods (logistic regression and random forest) in predicting AST. We showed that many of the applied modelling techniques were accurate in predicting susceptibility, with most of the model AUC-ROC values showing outstanding performance.

In logistic regression model, prior susceptibility to ceftriaxone was associated with a prediction of non-susceptibility to each of the three antibiotics. We postulate that this could be related to the nature of ceftriaxone as a third-generation cephalosporin. The use of broad-spectrum antibiotics is generally regarded as a driver for resistance.

The main advantage of the study was its sample size. The data of 26 087 cases with their complete electronic health record were included in the dataset. Patients from both community and hospitals were all recruited in the study, representing the entire spectrum of UTI encountered in clinical practice.

Another advantage of the study was the validity of the study. Model validation (train-test split) was performed on dataset obtained from a separate year. The model was trained with data of 2015–2019, and validation was performed on the data of 2020. This offered a prospective validation of the model. Regarding the lack of external validation, further studies in other sites will be required to validate utility and performance of this model.

The main predictive factors in our logistic regression models and predictive features with the highest SHAP values in our random forest models were all clinically relevant to the prediction to AST.

One drawback of the study was the large number of features required for model training. Given that the random forest models were trained with 105 features. However, many predictive models using big data are often integrated to the electronic health system as a clinical decision support tool. The models could be used to provide clinicians with valuable information on the likelihood of AMR in UTI in different patients. A future direction is to create a ‘lite’ version of the model for easier bedside calculation when immediate access to electronic health system is not available.

Another potential limitation with the study was the inclusion of only healthcare data in the public healthcare system in Hong Kong. As patient receive care in both public and private sectors locally, there would be an under-estimation of actual antimicrobial consumption.

New laboratory technologies have been described in the literature for rapid identification and susceptibility testing in urine culture. Common examples are MALDI-TOF, computer vision, fluorescence analysis and rapid AST.^13–15^ While they are valuable in shortening the specimen processing and reporting time, they cannot replace the judicious selection of empirical antibiotics treatment.

The aim of this AI model is to predict susceptibility categories for various commonly used first-line antibiotics for UTI. However, these antimicrobial agents are not without side effects. Nitrofurantoin use is contraindicated in renal failure, and ciprofloxacin has multiple black box warning. As an antimicrobial stewardship tool, this model can aid clinicians in predicting antibiotic susceptibility. But prescribers must also consider other factors during prescription. The development of AI models based on healthcare data to aid safe prescription would be another future direction.

Previous studies on AST prediction with various AI algorithms from different geographical areas^10,12,16,17^ showed that they were accurate. Studies that compared the predicted antimicrobial prescription and actual antimicrobial prescription also showed that AI algorithms could reduce the use of broad-spectrum antibiotics significantly.^10,16^ They show the powerful potential of AI in the field of antimicrobial stewardship.

### Conclusion

Random forest model may aid judicious empirical antibiotics use in UTI. Given the reasonable performance and accuracy, these accurate models may aid clinicians in choosing between different first-line antibiotics for UTI.

## Supplementary Material

dlae121_Supplementary_Data

## Supplementary data

Tables S1 and S2 are available as Supplementary data at *JAC-AMR* Online.
