# Supplementary material for: Predicting antibiotic susceptibility in urinary tract infection with artificial intelligence—model performance in a multi-centre cohort
Source: JAC Antimicrob Resist. 2024 Aug 7;6(4):dlae121. doi: 10.1093/jacamr/dlae121 (PMC11304604; doi:10.1093/jacamr/dlae121)
Supplement: dlae121_Supplementary_Data [file dlae121_supplementary_data.docx]

| Table S1 – Baseline characteristics of the study cohort (n=26087) | |
| --- | --- |
| Variable | Count (%) / Mean (SD) |
| **Basic epidemiological factors** | |
| Male gender | 8888 (34.1%) |
| Any use of immunosuppressant in the past 5 years | 2283 (8.75%) |
| Age in years | 67.9 (21.0) |
| **Antibiotics consumption in the past 5 years (defined daily dose)** | |
| Aminoglycosides | 0.59 (7.75) |
| Antifungal agent | 6.01 (72.73) |
| Beta-lactam beta-lactamase inhibitor combination | 40.71 (54.29) |
| Carbapenems | 2.89 (9.42) |
| 1^st^ generation cephalosporins | 0.32 (4.68) |
| 2^nd^ generation cephalosporins | 2.05 (10.73) |
| 3^rd^ generation cephalosporins | 1.62 (5.81) |
| 4^th^ generation cephalosporins | 0.13 (1.58) |
| 5^th^ generation cephalosporins | 0.01 (0.42) |
| Clindamycin | 0.1 (1.57) |
| Anti-CMV antiviral agents | 0.47 (12.74) |
| Colistin | 0.05 (1.24) |
| Anti-COVID antiviral agents | 0.07 (5.59) |
| Gram-positive agents (Vancomycin, Daptomycin, Linezolid) | 1.38 (10.3) |
| Anti-helminthic agents | 0.01 (0.67) |
| Antivirals against HSV and VZV | 2.68 (36.4) |
| Metronidazole | 2.9 (18.63) |
| Anti-influenza antivirals | 1.43 (4.02) |
| Macrolides | 2.48 (51.56) |
| Penicillins | 5.64 (34.85) |
| Quinolones | 10.22 (46.41) |
| Rifamycin | 4.52 (56.36) |
| Anti-tuberculosis medications other than Rifamycins | 6.24 (79.06) |
| Tetracyclines | 4.3 (58.04) |
| Nitrofurantoin | 7.54 (71.82) |
| **Blood test results in the previous 5 years** | |
| Haemoglobin maximum value (g/dL) | 12.8 (1.7) |
| Haemoglobin minimum value (g/dL) | 10.72 (2.45) |
| Haemoglobin mean value (g/dL) | 11.74 (1.86) |
| Haemoglobin standard deviation (g/dL) | 0.71 (0.62) |
| White blood cell count (WBC) maximum value (x 10^9/L) | 13.26 (7.64) |
| WBC minimum value (x 10^9/L) | 6.91 (3.6) |
| WBC mean value (x 10^9/L) | 9.32 (3.89) |
| WBC standard deviation (x 10^9/L) | 2.24 (2.35) |
| Platelet count maximum value (x 10^9/L) | 297.02 (128.32) |
| Platelet count minimum value (x 10^9/L) | 192.75 (80.42) |
| Platelet count mean value (x 10^9/L) | 237.93 (85.55) |
| Platelet count standard deviation (x 10^9/L) | 35.62 (36.73) |
| Creatinine maximum value (μmol/L) | 145.03 (169.42) |
| Creatinine minimum value (μmol/L) | 83.25 (65.45) |
| Creatinine mean value (μmol/L) | 106.45 (97.14) |
| Creatinine standard deviation (μmol/L) | 19.98 (42.83) |
| Bilirubin maximum value (μmol/L) | 18.27 (24.25) |
| Bilirubin minimum value (μmol/L) | 8.1 (8.05) |
| Bilirubin average value (μmol/L) | 12.08 (12.48) |
| Bilirubin standard deviation (μmol/L) | 3.71 (8.39) |
| Alanine transaminase maximum value (IU/L) | 53.64 (164.8) |
| Alanine transaminase minimum value (IU/L) | 16.74 (16.18) |
| Alanine transaminase average value (IU/L) | 27.94 (40.94) |
| Alanine transaminase standard deviation (IU/L) | 12.44 (47.9) |
| Alkaline phosphatase maximum value (IU/L) | 129.69 (139.04) |
| Alkaline phosphatase maximum value (IU/L) | 76.19 (45.31) |
| Alkaline phosphatase maximum value (IU/L) | 96.81 (68.09) |
| Alkaline phosphatase maximum value (IU/L) | 18.24 (37.9) |
| **Number of disease episodes in electronic health record in the past 5 years** | |
| Accident | 0.75 (1.12) |
| Atrial fibrillation | 0.15 (0.45) |
| Cancer | 0.42 (1.07) |
| Chest infection | 0.56 (0.99) |
| Congestive heart failure | 0.23 (0.64) |
| Chronic lung disease (other than infection) | 0.18 (0.59) |
| Congenital disorder | 0.04 (0.4) |
| Cardiovascular disorder (other than heart failure) | 0.63 (1.31) |
| Dermatological disorder | 0.23 (0.65) |
| Diabetes mellitus (DM) | 0.69 (1.63) |
| Endocrinological disorder (other than DM) | 0.79 (1.37) |
| Fracture | 0.23 (0.63) |
| Gastrointestinal tract disorder | 1.15 (1.84) |
| Genitourinary tract disorder | 1.02 (1.57) |
| Hyperlipidaemia | 0.29 (0.56) |
| Hypertension | 0.77 (0.88) |
| Ill-defined illness | 1.82 (2.24) |
| Number of episodes of infection | 0.91 (1.31) |
| Injury | 0.57 (1.07) |
| Pulmonary disorder | 0.23 (0.61) |
| Musculoskeletal disorder | 0.58 (1.23) |
| Neonatal disorder | 0.04 (0.43) |
| Obstetric condition | 0.27 (1.6) |
| Psychiatric condition | 0.97 (1.62) |
| Retention of urine | 0.58 (1.12) |
| Stroke | 1.07 (1.62) |
| Upper respiratory tract infection | 0.11 (0.35) |
| **Number of bacterial/fungal isolates in the past 5 years from all specimens** | |
| Methicillin-resistant *Staphylococcus aureus* | 0.11 (0.48) |
| Non-fermenters | 0.12 (0.61) |
| Third-generation cephalosporin-resistant Enterobacterales | 0.37 (0.95) |
| *Candida albicans* | 0.05 (0.39) |
| *Candida* spp. other than *C. albicans* | 0.03 (0.28) |
| Extended spectrum beta-lactamase | 18.13% |
| **Antimicrobial susceptibility in urinary tract pathogens in the past 5 years (percentage susceptible)** | |
| Nitrofurantoin | 76.54% |
| Amoxicillin-clavulanate | 68.55% |
| Meropenem | 91.03% |
| Ciprofloxacin | 62.82% |
| Amikacin | 99.53% |
| Cefuroxime | 65.99% |
| Cefotaxime / Ceftriaxone | 70.31% |
| Vancomycin | 99.96% |

| Table S2 – Precision-recall curves of random forest models | | |
| --- | --- | --- |
| Nitrofurantoin | Amoxicillin-clavulanate | Levofloxacin |
| 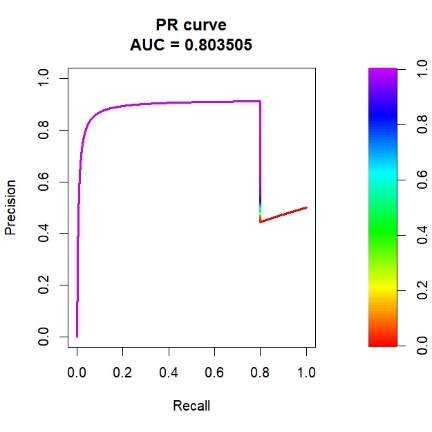 | 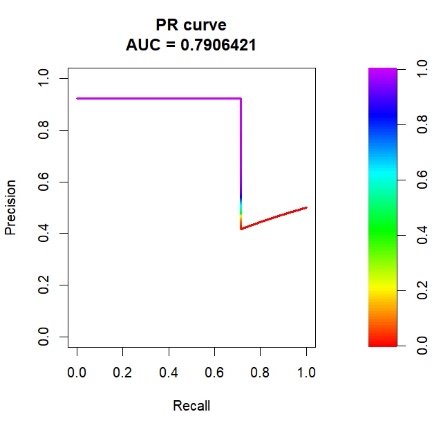 | 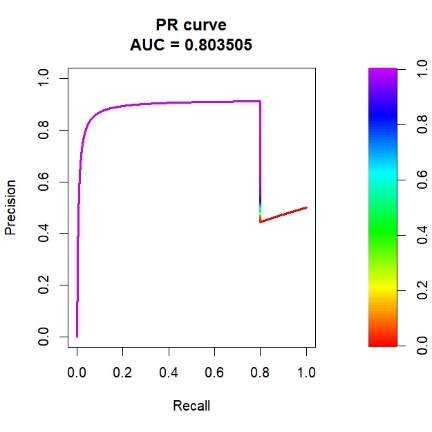 |

| Table S3a – confusion matrix for nitrofurantoin using random forest | | | |
| --- | --- | --- | --- |
|  |  | Predicted | |
| Actual |  | Resistant | Susceptible |
|  | Resistant | 947 | 180 |
|  | Susceptible | 396 | 4327 |

| Table S3b – confusion matrix for amoxicillin-clavulanate using random forest | | | |
| --- | --- | --- | --- |
|  |  | Predicted | |
| Actual |  | Resistant | Susceptible |
|  | Resistant | 1238 | 380 |
|  | Susceptible | 241 | 3991 |

| Table S3c – confusion matrix for ciprofloxacin using random forest | | | |
| --- | --- | --- | --- |
|  |  | Predicted | |
| Actual |  | Resistant | Susceptible |
|  | Resistant | 1813 | 295 |
|  | Susceptible | 398 | 3344 |

| Table S3d – confusion matrix for nitrofurantoin using logistic regression | | | |
| --- | --- | --- | --- |
|  |  | Predicted | |
| Actual |  | Resistant | Susceptible |
|  | Resistant | 824 | 303 |
|  | Susceptible | 229 | 4494 |

| Table S3e – confusion matrix for amoxicillin-clavulanate using logistic regression | | | |
| --- | --- | --- | --- |
|  |  | Predicted | |
| Actual |  | Resistant | Susceptible |
|  | Resistant | 1263 | 355 |
|  | Susceptible | 263 | 3969 |

| Table S3f – confusion matrix for ciprofloxacin using logistic regression | | | |
| --- | --- | --- | --- |
|  |  | Predicted | |
| Actual |  | Resistant | Susceptible |
|  | Resistant | 1808 | 300 |
|  | Susceptible | 435 | 3307 |
